# The detection of *pfcrt* and *pfmdr1* point mutations as molecular markers of chloroquine drug resistance, Pahang, Malaysia

**DOI:** 10.1186/1475-2875-11-251

**Published:** 2012-08-01

**Authors:** Wahib M Atroosh, Hesham M Al-Mekhlafi, Mohammed AK Mahdy, Johari Surin

**Affiliations:** 1 Department of Parasitology, Faculty of Medicine, University of Malaya, Kuala Lumpur 50603, Malaysia; 2 Department of Parasitology, Faculty of Medicine and Health Sciences, Sana’a University, Sana’a, Yemen

## Abstract

**Background:**

Malaria is still a public health problem in Malaysia with chloroquine (CQ) being the first-line drug in the treatment policy of uncomplicated malaria. There is a scarcity in information about the magnitude of *Plasmodium falciparum* CQ resistance. This study aims to investigate the presence of single point mutations in the *P. falciparum* chloroquine-resistance transporter gene (*pfcrt*) at codons 76, 271, 326, 356 and 371 and in *P. falciparum* multi-drug resistance-1 gene (*pfmdr1*) at codons 86 and 1246, as molecular markers of CQ resistance.

**Methods:**

A total of 75 *P. falciparum* blood samples were collected from different districts of Pahang state, Malaysia. Single nucleotide polymorphisms in *pfcrt* gene (codons 76, 271, 326, 356 and 371) and *pfmdr1* gene (codons 86 and 1246) were analysed by using mutation-specific nested PCR and restriction fragment length polymorphism (PCR-RFLP) methods.

**Results:**

Mutations of *pfcrt* K76T and *pfcrt* R371I were the most prevalent among *pfcrt* gene mutations reported by this study; 52% and 77%, respectively. Other codons of the *pfcrt* gene and the positions 86 and 1246 of the *pfmdr1* gene were found mostly of wild type. Significant associations of *pfcrt* K76T, *pfcrt* N326S and *pfcrt* I356T mutations with parasitaemia were also reported.

**Conclusion:**

The high existence of mutant *pfcrt* T76 may indicate the low susceptibility of *P. falciparum* isolates to CQ in Peninsular Malaysia. The findings of this study establish baseline data on the molecular markers of *P. falciparum* CQ resistance, which may help in the surveillance of drug resistance in Peninsular Malaysia.

## Background

Anti-malarial drug resistance is a major challenge to the control of falciparum malaria, the leading cause of morbidity and mortality especially in Africa and southern Asia [1]. The first *P. falciparum* chloroquine resistance was reported in the late 1950s in Southeast Asia along the Thai-Cambodian border [2,3]. Further spread of CQ resistance was shown later to include neighboring countries in Asia, South America and Africa [4-8]. Moreover, *P. falciparum* has been also reported resistant to other anti-malarial drugs including sulphadoxine/pyrimethamine drug combination, mefloquine, atovaquone and artemisinin [9-12].

Three methods have been commonly used for monitoring anti-malarial drug efficacy and to screen for drug resistance; *in vivo* test, *in vitro* test and, more recently, the detection of molecular markers of anti-malarial drug resistance [13]. CQ resistance has been associated with point mutations in two genes; *P. falciparum* chloroquine resistance transporter (*pfcrt*) and multidrug resistance 1 (*pfmdr1*). It is well documented that the mutation at codon 76 of the *pfcrt* gene (*pfcrt* K76T), resulting in the substitution of threonine for lysine at position 76, is a key marker of *P. falciparum* CQ resistance [14]. However, the role of *pfmdr1* point mutations on CQ resistance *P. falciparum* isolates remains a matter of debate.

In Malaysia, malaria is still a public health problem especially in the interior parts of the Peninsula and the states of Sabah and Sarawak (East Malaysia), with *P. falciparum* and *Plasmodium vivax* being the predominant species. A previous *in vivo* study followed by a molecular detection of resistance markers has been conducted in Sarawak and showed that CQ resistance occurred widely and therefore, CQ was replaced by sulphadoxine/pyrimethamine (SDX/PYR), and later by artemisinin combination therapy, as the drug of choice for treating uncomplicated falciparum malaria infections in East Malaysia [15]. In West Malaysia, CQ is still used as the first-line drug in the treatment policy of uncomplicated malaria caused by *P. falciparum*, although a previous *in vivo* study reported a CQ resistance rate of 63.6% among uncomplicated falciparum malaria patients [16]. Data on the molecular markers of CQ resistance in Peninsular Malaysia is not available. Therefore, this study was carried out to detect the molecular markers of anti-malarial drug resistance based on *P. falciparum* chloroquine resistance transporter (*pfcrt*) and *P. falciparum* multidrug resistance 1 (*pfmdr1*) genes.

## Methods

### Study area

This study was carried out in Pahang state which is the largest state in Malaysia after Sabah and Sarawak (East Malaysia). The state is divided into 11 administrative districts and is located in the eastern North Kuala Lumpur with an area of 36,137 km^2^ and a total population of 1,443,365. The climate is equatorial with an average temperature of 21-32°C and an annual rainfall of between 2,000- 2,500 mm. Relative humidity in such areas is in the range of 80%. The economy is mostly agricultural; palm oil, natural rubber and timber are the major primary commodities. Malaria is endemic in Pahang, with *P. falciparum* and *P. vivax* accounting for the majority of reported cases.

### Sampling and malaria microscopy

**Figure 1:**
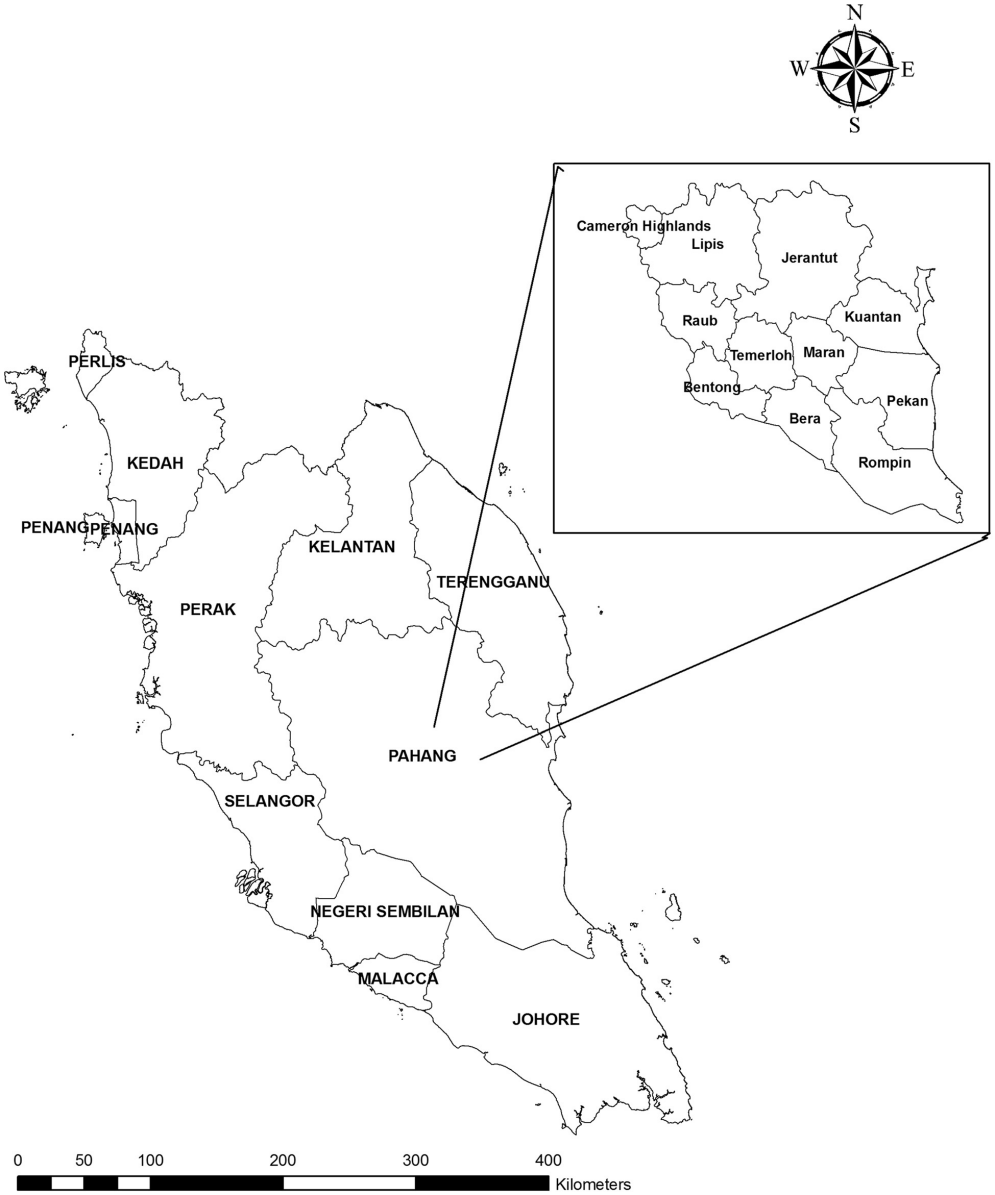
A geographic map shows Peninsular Malaysia and Pahang’s districts.

A total of 822 blood samples were collected from the different districts of Pahang (728 survey-based blood samples and 94 archived positive falciparum malaria slides) (Figure 1). The survey was conducted between May 2010 and May 2011 while the archived slides are for cases reported between 2007 and 2009. During the survey, finger prick blood samples were collected from participants in 20 villages of different districts of Pahang, and thick and thin blood films were prepared. Filter paper blood spots were also collected from each participant on 3MM Whatman® filter paper (Whatman International Ltd., Maidstone, England), and kept in clean, dry and well-sealed separate plastic bags for molecular analysis. Demographic data of the participants were collected by a standard semi structural questionnaire throughout face-to-face interview during the sampling survey. For archived slides, data was collected from the patients’ records.

Thick and thin blood films were stained with 10% diluted Giemsa and examined microscopically for the presence of malaria. About 200 fields under 1,000x magnification were examined from the thick film before the slide was considered negative. Parasitaemia was calculated by counting the asexual stages of the parasite against 300 white blood cells (WBC) and multiplied by 7500 as an assumed average total WBC count for the individuals [17]. Parasitaemia was categorized as low (< 1,000 parasites/μl of blood), moderate (1,000 – 9,999 parasites/μl of blood) and high (≥ 10,000 parasites/μl of blood). Archived malaria positive slides were also re-examined and parasitaemia were recorded.

### Molecular identification of *Plasmodium* species

Genomic DNA was extracted from filter papers blood spots and from archived blood smears. Briefly, a disc of the filter paper blood spot (approximately 6 mm diameter) was punched out using a methanol-flamed puncher and placed in 1.5 ml centrifuge tubes using clean and flamed forceps. Archived slides were first cleaned with chloroform to remove oil and 50 ml of TE buffer was transferred onto the smear. The smear was completely wiped off the slide using small Whatman filter paper disc (cut by flamed-sterile puncher) and transferred into 1.5 ml microcentrifuge tubes. Genomic DNA was extracted using Qiagen blood and tissue kit (QIAGEN, DNeasy® Blood & Tissue Kit, Cat. no. 69506, Germany) according to the manufacurer’s instruction. DNA extract was finally eluted using 50 μL Qiagen AE elution buffer and kept at −20°C until used. *Plasmodium* species were identified using genus-specific and species-specific primers in nested PCR based on small subunit ribosomal RNA (18 s rRNA) gene [18]. PCR reaction and cycling conditions were conducted as mentioned previously [19].

### Molecular detection of *pfcrt* and *pfmdr1* mutations

The detection of mutations of different positions of *pfcrt* and *pfmdr1* genes was performed using mutation-specific nested PCR and restriction fragment length polymorphism (PCR-RFLP) according to the protocols described previously [14,20].

Primers for *pfcrt* K76T primary amplification were TCRP1 and TCRP2 while the secondary PCR was conducted by using the forward primer CRTP3 and the reverse primers TCRP4 m and TCRP4 w for mutant and wild types, respectively. *Pfcrt* Q271E alleles were amplified using primer set of CRT2a and CRT2b in the primary PCR and CRT271a and CRT271b in the secondary PCR. Amplification of N326S, I356T and R371I *pfcrt* mutations was carried out using the same primer set (CRT-3a and CRT-3b) in the first reaction, while secondary reactions were done using CRT326a and CRT326b, CRT356a and CRT356b, and CRT371a and CRT371b, respectively. A 20 μl reaction volume containing 8 μl of the secondary PCR amplicons and 1 unit of specific endonuclease digestion enzyme was incubated overnight at 37°C. The restriction enzymes XMN1, MSe1, AlwN1 and AflII were used for the digestion of *pfcrt* 271, 326, 356 and 371 amplicons while the mutant *pfcrt* allele T76 remained uncut.

For the detection of *pfmdr1* gene mutations (N86Y and D1246Y)**,** primers sets (MDR-A and MDR-B) and (1246-A and 1246-B) were used for the primary amplification of *pfmdr1* 86 and *pfmdr1* 1246, respectively while the secondary PCR was conducted using primers sets (MDR-D1 and MDR-D2) and (1246-D1 and 1246-D2) for flanking the mutation site of *pfmdr1* 86 and *pfmdr1* 1246 codons, respectively. Then, the *Pfmdr1* N86Y and D1246Y amplicons were digested using Af1III and Bg1II restricted enzymes, respectively. The primers and PCR reagents were from iNtRON (iNtRON Biotechnology, Inc. Seoul, Korea). All PCR products were resolved by electrophoresis in a 2% agarose gel stained with SYBR safe and visualized by UV-transilluminator.

Genomic DNA of HB3, 3D7 and Dd2 strains of *P. falciparum* provided by Malaria Research and Reference Reagents Resources Centre (MR4, ATCC®, Manassas VA, USA) were used as positive controls for wild and mutant types, respectively.

### Ethical consideration

The protocol of this study (Reference Number: 788.73) has been approved by the Medical Ethics Committee of the University of Malaya Medical Centre, Kuala Lumpur, Malaysia. The study was also registered with the National Medical Research Registry, Malaysia (Research ID: 5681). Before the commencement of sampling, the objectives of the study were explained to the people in their village setting and consents were obtained from those who have agreed to participate in the study.

### Statistical analysis

Data was analysed using the SPSS statistical package version 13. Statistical associations between point mutations and parasitaemia were assessed using the Chi-square test with Yates' continuity correction. When the number of expected observations in one or more cells in a 2 × 2 contingency table is less than 5, Fisher’s Exact test was used. A p value ≤ 0.05 was considered statistically significant.

## Results

Of the 822 collected samples, 728 were collected by sampling surveys in 20 villages of different districts of Pahang, and 94 were archived malaria positive slides from different health centres, hospitals and malaria control units in the same study area. *Plasmodium falciparum* parasites were detected in 75 of the total collected samples (64 from archived slides and 11 from surveys). Based on these 75 slides, the mean age of participants was 27.8 ± 4.1 years and 66% were males. The majority of participants were Malay (64.2%) followed by Aborigines (23.9), Indians (6.5%) and Chinese (2.7%). Moreover, 2 slides (2.7%) were from foreign workers who were residing in Malaysia for the past three years.

*Plasmodium falciparum* parasite density ranged from 50 asexual parasites/μl of blood to 17,100 with a parasitaemia geometric mean of 4,065 asexual parasites/μl. Moderate-to-high parasitaemic individuals (parasite count ≥ 1,000 parasites/μl of blood) were more frequent 58.7% while individuals with low parasitemia represented 41.3%.

**Figure 2:**
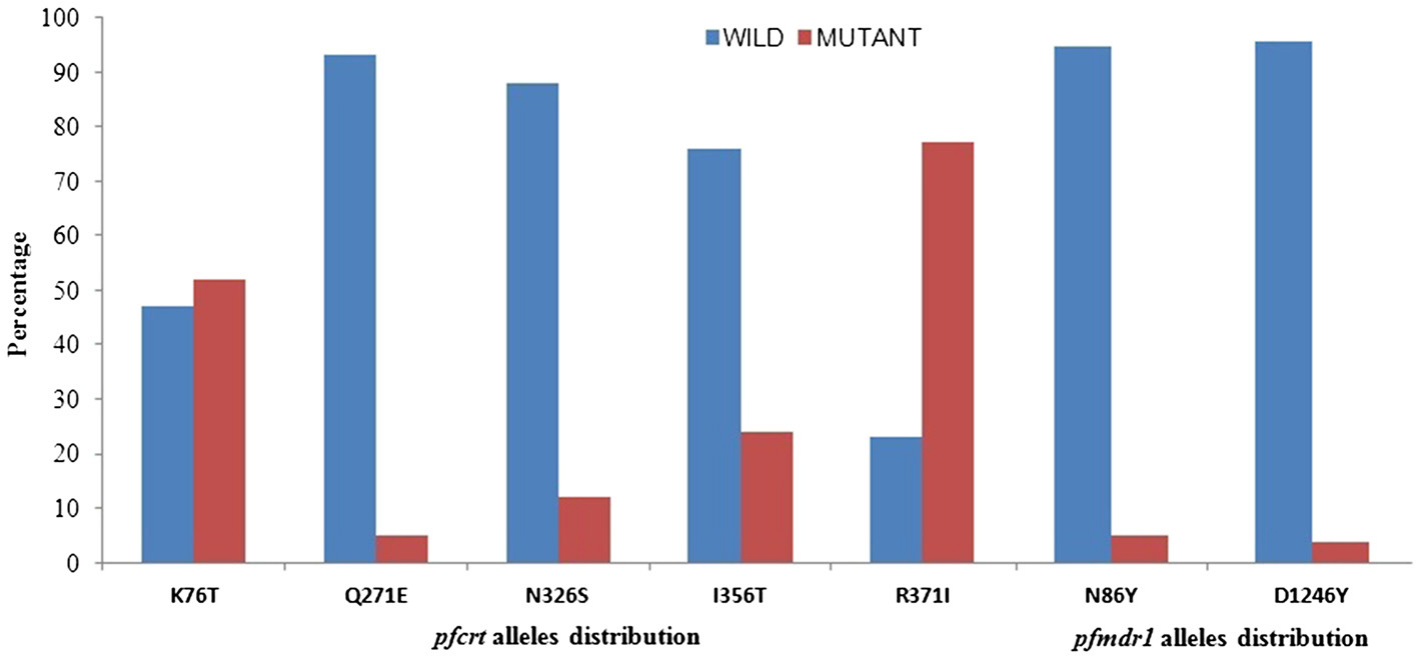
**Distribution of *pfcrt *and *pfmdr1 *alleles in *Plasmodium falciparum *isolates from Pahang, Malaysia.**

All falciparum positive slides were examined for single nucleotide polymorphisms (SNP) at five positions of *pfcrt* gene (K76T, Q271E, N326S, I356T and R371I) and two positions of *pfmdr1* genes (N86Y and D1246Y), and the results are shown in Figure 2. The detection rate was 100% for all positions of *pfcrt* and *pfmdr1* markers except for 76 and 271 position of *pfcrt* (98.7%); one sample was negative for each of them. Of *pfcrt* point mutations, *pfcrt* K76T and *pfcrt* R371I had higher mutant alleles than other positions (52% and 77%, respectively). Other positions showed predominance for the wild type with rates of 93%, 88% and 76% for the *pfcrt* Q271, *pfcrt* N326 and *pfcrt* I356, respectively. Moreover, polymorphism analysis of *pfmdr1* revealed a high predominance of wild type for both markers; 95% and 96% for *pfmdr1* N86 and *pfmdr1* D1246, respectively.

The association of mutations at different codons of *pfcrt* and *pfmdr1* with the parasitaemia, age of participant, gender, race and year of infection was examined (Table 1). Of the five positions of mutation at *pfcrt* gene, mutations at codon 76, 326 and 356 showed significant associations with moderate-to- high parasitaemia as compared to low parasitaemia (p < 0.01). On the other hand, the association of *pfcrt* mutations with age, race, gender of participants and year of infection was not significant (p > 0.05). Similarly, the association of *pfmdr1* gene mutations at positions 86 and 1246 with the tested variables was not significant.

**Table 1:** **Association between parasitaemia and different point mutations of *pfcrt *and *pfmdr1 *genes**

**Locus**	**Alleles**	**Parasitaemia**		***χ*2 significance**
		**Low (< 1,000/μl)**	**Moderate to high (≥1,000/μl)**	
***Pfcrt *76**	Mutant	8	31	<0.001
	Wild	23	13	
***Pfcrt *271**	Mutant	0	4	0.138^*^
	Wild	31	40	
***Pfcrt *326**	Mutant	0	9	0.009^*^
	Wild	31	35	
***Pfcrt *356**	Mutant	1	17	<0.001^*^
	Wild	30	27	
***Pfcrt *371**	Mutant	22	36	0.409
	Wild	9	8	
***Pfmdr1 *-86**	Mutant	0	4	0.138^*^
	Wild	31	40	
***Pfmdr1 *-1246**	Mutant	0	3	0.263^*^
	Wild	31	41	

^*^ Fisher’s Exact test.

## Discussion

This is the first study in Peninsular Malaysia on the detection of molecular markers of *pfcrt* and *pfmdr1* for chloroquine and other anti-malarial drugs resistance. The high prevalence rate (52%) of *pfcrt* K76T point mutation reported by the present study reflects low susceptibility of *P. falciparum* to the CQ. This is in agreement with previous studies in Malaysia and neighbouring countries including Thailand, Indonesia and Philippine [15,16,21-23]. In Sarawak (East Malaysia), Cox-Singh *et al.* reported a strong association of *pfcrt* K76T with *in vitro* CQ resistance; *pfcrt* K76T was found higher at a fixation level (100%) in all isolates resistant to CQ [15]. Similar detection rates of mutant *pfcrt* T76 with a significant association with *in vitro* CQ resistance had also been reported in Thailand and Philippine [21,23].

Previous studies in other countries have showed such association between *pfcrt* K76T mutation and *in vivo* resistance of *P. falciparum* to CQ. In Mali, the presence of *pfcrt* K76T mutation was reported in all infections occurred within two weeks of administration of chloroquine while it was absent in all isolates that responded well to CQ [14,24]. Similar findings were reported in Uganda, Sudan and Yemen [19,25,26]. In the same vein, a previous study aimed at comparing two areas with different susceptibility levels to CQ found that the prevalence of wild type of *pfcrt* K76 was higher in areas with lower CQ resistance, while the mutant type was higher in areas with higher CQ resistance [27].

Except for *pfcrt* 371, the findings showed that other examined codons of the *pfcrt* gene (271, 326 and 356) were found mostly of wild type represented 93%, 88% and 76%, respectively. Moreover, *pfcrt* R371I mutation was detected in 77% of the isolates. Previous reports had suggested a correlation between CQ resistance and the accumulation of point mutations in the *pfcrt* gene [28,29]. However, the role of these mutations in conferring CQ resistance is unclear and suggested to have a complementary effect in maintaining the functional property of transporter protein [14,30]. Thus, further studies to explain the role of these mutations including *pfcrt* R371I are required.

Regarding *pfmdr1*, the present study showed a predominance of wild type of both *pfmdr1* N86 and *pfmdr1* D1246 (95% and 96%, respectively). The role of *pfmdr1* gene mutations in anti-malarial drugs resistance is still controversial. Previous *in vivo* studies reported the absence of mutations at codons *pfmdr1* 86 and 1246 in the CQ resistant infections [31-33]. Similarly, a previous study in south-eastern Iran reported a strong association between *pfcrt* K76T, but not *pfmdr1* N86Y mutation and *in vivo* CQ resistance [34]. In contrast, a recent study from Madagascar reported an association between *pfmdr1* Y86 mutant alleles and CQ clinical resistance with no such association with *pfcrt* gene [35]. Moreover, an *in vitro* study showed that *pfmdr1* mutations in *P. falciparum* can confer resistance to high levels of chloroquine, and that these *pfmdr1* mutations has an important role in the resistance of *P. falciparum* to mefloquine and quinine [36].

The high prevalence of wild alleles of *pfmdr1* N86 and *pfmdr1* D1246 could be considered as an indicator for low susceptibility of *P. falciparum* isolates to mefloquine, amodiaquine and quinine [21,37-40]. On the other hand, previous studies have found increased sensitivity to the anti-malarials mefloquine and artemisinin in *P. falciparum* isolates with mutations in the *pfmdr1* gene [41,42]. However, a previous *in vivo* study in Thailand found that treatment failure with mefloquine is significantly associated with increased *pfmdr1* copy number but not with the single point mutations [43].

The present study found a significant association between moderate-to-high parasitaemia and *pfcrt* point mutations at codons 76, 326 and 356. This is in agreement with previous studies in Tanzania, Sudan, Nigeria and Yemen [19,44,45]. However, studies from Sudan and Gabon showed no significant association between the *pfcrt* point mutations and severity of malaria [46,47]. Moreover, a recent study in the Yemen found higher frequency of *pfcrt* K76T mutation in *P. falciparum* isolates from patients aged > 10 years as compared to younger individuals [19]. On the other hand, a previous study among Nigerian children aged ≤ 12 years with acute uncomplicated *P. falciparum* malaria showed that the association between the presence of both mutant *pfcrt* T76 and *pfmdr1* Y86 alleles with *in vivo* anti-malarial drug resistance was age dependent [38]. However, the present study found no significant association between gene point mutations and age of patients. Most of the positive samples in this study were from adult patients aged 22–40 years, and this limited range of age may constraint examining such association.

## Conclusions

The high prevalence of mutation at codon 76 of the *pfcrt* gene (*pfcrt* K76T) is a key indicator of *P. falciparum* CQ resistance spread in Peninsular Malaysia. A further study to examine the implemented malaria drug policy using PCR-corrected anti-malarial drug efficacy trail in large sample set nationally is recommended. Continuous molecular surveillance using *pfcrt* and *pfmdr1* genes as molecular markers of chloroquine resistance are highly recommended in both East and West Malaysia, for monitoring the efficacy of chloroquine in Peninsular Malaysia and for the re-emergence of wild-type of *P. falciparum* in East Malaysia (Sarawak and Sabah).

## Competing interests

The authors declare that they have no competing interests.

## Authors’ contributions

WMA was involved in all phases of the study, including study design, data collection, data analysis, interpretation, and write-up of the manuscript; HMA, MAKM and JS designed the study; WMA and HMA performed the statistical analysis; MAKM provided technical advisory support in genotyping and data interpretation; All authors read and approved the final manuscript.
